# Transluminal Angioplasty of Transplanted Renal Artery Stenosis: A Review of the Literature for Its Safety and Efficacy

**DOI:** 10.1155/2011/693820

**Published:** 2011-04-14

**Authors:** Polytimi Leonardou, Sofia Gioldasi, Paris Pappas

**Affiliations:** Department of Radiology, Laikon General Hospital of Athens, 17 Ag. Thoma Street, 115 27 Athens, Greece

## Abstract

Transplant renal artery stenosis (TRAS) is a well-known cause of posttransplant hypertension accompanied by possible graft dysfunction and is potentially curable when is diagnosed early. Colour Doppler Ultrasonography (CDU) is the screening procedure of choice in most studies whereas some centers employ Magnetic Resonance Angiography (MRA), if available. Although both CDU and MRA can arouse suspicion of disease in less symptomatic cases, angiographic techniques are essential for confirmation of TRAS. Percutaneous Transluminal Angioplasty (PTA) is a good and widespread therapeutic approach for the treatment of TRAS due to its acceptable complication rate and high technical success rate. The purpose of this paper is to assess the safety and efficacy of PTA in the treatment of TRAS, to compare the long-term outcomes between different reports, and to examine the role of PTA with stenting in inhibiting recurrence of the disease.

## 1. Introduction

Transplanted renal artery stenosis (TRAS) is the most common vascular complication of transplanted kidney, which causes refractory hypertension and can result in allograft dysfunction which presents with increase of serum creatinine level. Transplanted renal artery stenosis (TRAS) usually becomes apparent from 3 months to 2 years after transplantation [1]. In relation to arterial anastomotic site, the stenosis can be proximal because of recipients' atherosclerotic arterial disease, anastomotic due to surgical trauma in combination with postoperative fibrosis, and distal whose aetiology is not well defined, though mechanical and immunological factors are implicated as possible causes [2]. Percutaneous transluminal angioplasty and stenting (PTAS) has as main purpose the amelioration of graft function and blood hypertension. 

## 2. Angioplasty and Stenting in Transplant Renal Artery Stenosis: Technical and Clinical Aspects

There is a wide range of incidence of TRAS varying from 1.8% to 25% according to the literature, indicating the significant role of surgical team as well as the importance of the chosen screening procedure [3]. Clinical suspicion of TRAS is raised by the presentation of refractory hypertension and the increase of serum urea and creatinine levels. Colour Doppler Ultrasonography (CDU) is the most common method used for the detection of renal artery stenosis, because it is relatively inexpensive and harmless without ionizing radiation, even though its diagnostic value depends thoroughly on operator's experience and skill. Although both Magnetic Resonance Angiography (MRA) and Spiral Computed Tomography Angiography (CTA) provide three-dimensional image of the vascular tract, MRA is superior to CTA with higher sensitivity and specificity, consisting a viable technique for monitoring transplanted kidney's function; however the nephrotoxicity of contrast medium used remains a limitation for imaging the transplanted kidney with impaired function by tomographic methods. MRA with the intravenous Gadolinium contrast medium administration is the method of choice in cases of no impaired renal function. However, in patients with severe kidney failure it carries the risk of a possible nephrogenic systemic fibrosis as a complication. CTA is not usually indicated, because of the iodine contrast nephrotoxicity in high doses. Yet, for confirmation of TRAS selective percutaneous transluminal angiography, which needs low-contrast medium quantities, is still considered as the gold standard [2–5].

 Three different treatment modalities are available for patients with TRAS: (i) medical management is indicated if the degree of stenosis is not considered hemodynamically or clinically very significant (<50%) and/or renal function has not been deteriorated significantly; (ii) surgical revascularization is indicated for cases of unsuccessful PTA or when the stenosis is not accessible to PTA; (iii) PTA accompanied by stent implantation (PTAS) when necessary is a less invasive procedure and represents the preferred option even when stenosis is in hilar or distal portion of the arterial renal bed.

 The procedure of PTAS is well defined and consists of the following steps.

**Figure 1:**
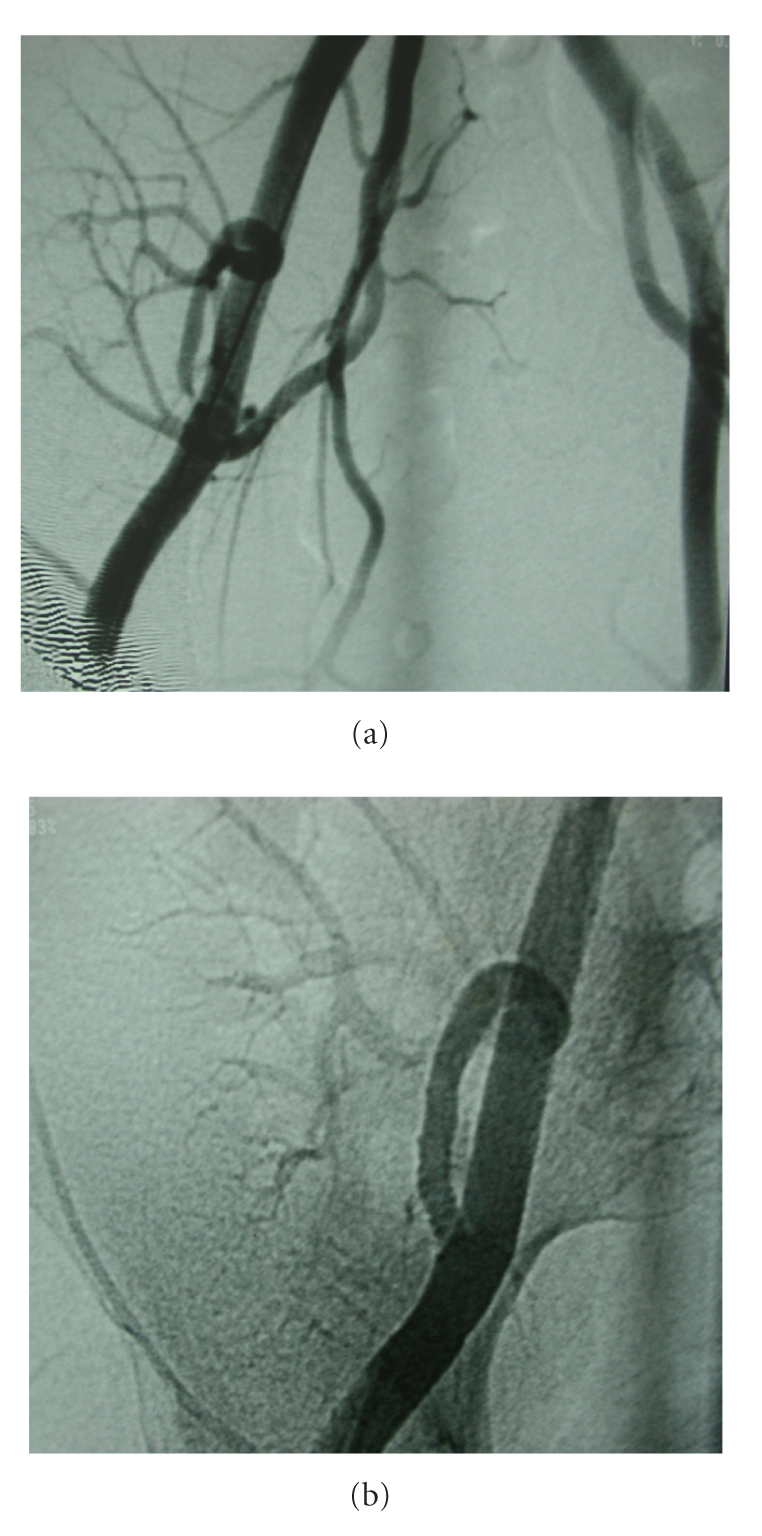
(a) Anastomotic transplant renal artery stenosis. (b) Postpercutaneous transluminal angioplasty and stenting image.

**Figure 2:**
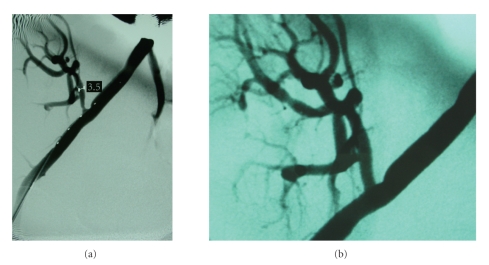
(a) Transplant renal artery stenosis. (b) Postpercutaneous transluminal angioplasty and stenting image.

**Figure 3:**
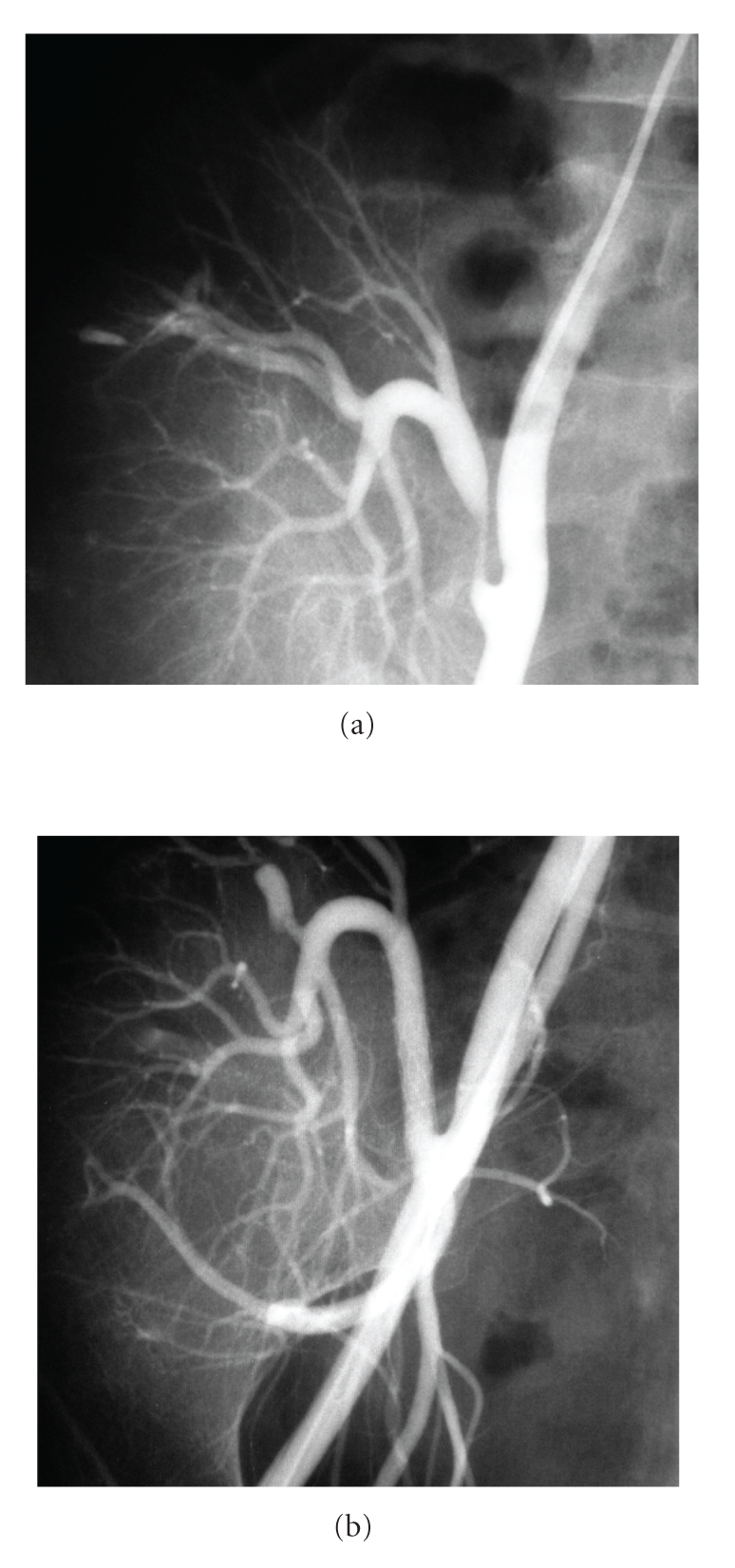
(a) Transplant renal artery stenosis and postbiopsy arteriovenous fistula. (b) Postpercutaneous transluminal angioplasty and stenting and post-embolization image.

1. Femoral artery approach is generally preferable (brachial or axillary ones can rarely be used in case of renal artery originating at close angle downward). An arterial sheath of 4F up to 6F is placed in the iliac artery, and then a guide wire is passed beyond the area of the artery that is going to be treated.
2. A preliminary angiographic study of the iliac arteries in anteroposterior and oblique projections is the initial, in order to localize the origin of renal artery and to visualize the site and morphology of the lesion (Figures 1(b), 2(b), and 3(b)).
3. After crossing stenosis with a soft guide wire, it is then replaced by a stiffer one to support balloon catheter. Heparin in doses of 5000 IU is also used to inhibit thrombotic complications. Spasm of the arteries can be reversed by administrating vasodilators.
4. The balloon catheter is placed across the stenotic area after angiographic control. The size of the balloon is defined by the diameter of the normal adjacent area of the renal artery. Then the balloon is inflated with a pressure comprised of about 5 to 10 atmospheres for about one minute, depending on segment's resistance. The above procedure can be repeated two or three times, if it is necessary.
5. The commonly used stents in PTA are balloon expandable and are crimped onto a same diameter balloon with this one used for previous angioplasty. This assembly is passed over the guide wire across the lesion, is inflated in correct position, and is then withdrawn leaving the stent in place. Postprocedural angiography is performed to ensure its position and suitability (Figures 1(b), 2(b), and 3(b)). Technical success is considered when the residual stenosis does not exceed 30% of the transplant renal artery normal diameter.
6. Hemostasis is achieved usually by manual compression, or, otherwise, a system of rapid hemostasis can be used in special circumstances.
7. Within 24–48 hours the clinical condition of the patient is estimated considering blood pressure, urine, and serum enzymes as parameters. Drop of the hypertension and serum urea and creatinine levels indicate signs of clinical success.

## 3. Safety and Efficacy of Percutaneous Transluminal Angioplasty and Stenting in Transplant Renal Artery Stenosis

Many reports in the literature address the role of PTA in patients with TRAS based upon its acceptable complication rate and its high technical and clinical success rates (reported as 65–100%) (Table 1) [4–17]. Indeed the majority of surveys show few acute complications and achievement of prompt correction of the stenosis. Pappas et al. [8] made a retrospective analysis of 22 patients with TRAS, all cases confirmed by angiography. All patients were handled by PTA with stenting immediately after the intra-arterial angiography. The technical success rate—elimination of stenosis—was 100%, no acute complications were verified, and amelioration of arterial hypertension and improvement of graft function were achieved within the first 7 postoperative days. In a follow-up period ranging from 3 to 104 months, two of 22 patients died from irrelevant causes with no graft function impairment, two experienced renal transplant failure (6 and 20 months after PTA), and the remaining 18 patients had good graft function with decreased blood pressure and decreased creatinine blood levels. Authors prefer PTA and PTAS as initial treatment of TRAS underlining the safety and efficacy of these procedures and suggest that surgery should be restricted to patients with failed PTA or with stenosis inaccessible to PTA due to its high risk of complications.

 In most recent reports a few major procedure-related complications occur (range from 0% to 10%), in contrast to surgery which is associated with a 15% graft loss rate and 5% mortality rate, reflecting the gained experience and sufficient equipment that have improved means of PTA [6, 18, 19]. Only in a large and long-standing (within 24-year time period) retrospective analysis by Peregrin et al. [9] complications were observed in 25.5% (13/51) of procedures. Specifically, three patients underwent kidney function impairment due to contrast medium nephrotoxicity leading to graft failure in one of them, while in the other two impairment was reversible; groin hematoma and pseudoaneurysm happened in four patients, all of them treated successfully percutaneously; dissection of the dilated lesion occurred in five patients, treated with stent implantation without functional deterioration of graft; one patient experienced peripheral branch occlusion without any clinical sequel.

 Regarding long-term outcomes, the assumption of investigation of the literature is promising. In several studies a significant decrease in serum creatinine as well as in blood pressure is underlined, and in some cases there was also decrease of the antihypertensive doses. In particular, in a trial by Bertoni et al. [20] of nine patients with TRAS undergone PTA, technical success was 100%, and arterial hypertension was controlled in seven patients out of nine and remarkably after one year from stenting seven patients remained normotensive and medication-free. On the other hand, PTA results may vary according to the duration of follow-up period. A graft survival rate of 95% after one year and 82% after two years has been reported [21]. Moreover, there is a variety of different parameters used in different studies to determine the beneficial effect of PTA on graft function that are not constantly reported so that comparison between studies becomes obscure. 

 Another matter of interest is whether PTA can affect restoration of transplanted kidney function. In a relatively recent study by Peregrin et al. [9], 6 out of 12 patients who experienced graft failure or were in dialysis programme days to weeks before PTA improved graft function for at least 6 months so that these patients could remain dialysis-free for some period of time. On the other hand, three patients who underwent graft failure and unsuccessful PTA started dialysis within days after PTA. In the same survey transplanted patients were divided to subgroups based on the clearance of creatinine (Ccr) and the comparison between those with impaired graft function (range of Ccr from 0.2 to 0.5/1.73 m^2^/(mL s)) and those with Ccr above 0.5/1.73 m^2^/(mL s) and revealed no significant difference in graft survival confirming and supporting the idea that successful PTA can reinforce graft function. 

 If PTA alone, the procedure carries higher risk of restenosis with a recorded incidence from 10% to 33% of cases over 6 or 8 months and from 16% to 62% of cases in general [13]. Many recent reports highlight the crucial role of PTA with stenting in limitation of recurrence, with success rate between 90 to 100% [22]. Remarkably, as discussed in a study by Salvadori et al. [14], the 5-year actuarial survival rate was similar in patients with TRAS treated with PTA and stenting (76.3%) versus patients who had also undergone renal transplantation and had not developed TRAS (72.9%). 

Based on these promising documents some authors suggest stent implantation already at first angioplastic procedure. In addition, the use of radioactive stents or stents that release antiproliferative agents to inhibit intimal hyperplasia can decrease the incidence of recurrence further more. 

## 4. Conclusion

In conclusion, PTA alone or in combination with stent implantation is a well-established technique and is also the initial interventional procedure of choice for high grade TRAS, due to its lower invasiveness and less severe complications, which overweigh the small possibility of noneffectiveness. Surgical revascularization is indicated if PTA cannot be done or if it is unsuccessful. Undoubtfully, close collaboration between nephrologist, surgeon and interventional radiologist is required aiming the adoption of the most suitable procedure for each patient.

## Figures and Tables

**Table 1:** Studies published over the last decade presenting transplant renal artery stenosis treated by percutaneous transluminal angioplasty and stenting.

Study (year)	Number of patients	Technical success (%)	Clinical Success (%)*	Follow-up (mean; months)	Complications related to the procedure (%)
Ghazanfar et al. (2011) [4]	44	100	86	60	—**
Saratnahaei et al. (2010) [5]	9	100	77.7	17.3	—
Henning et al. (2009) [6]	13	92.3	Not mentioned	33.15	—
Hagen et al. (2009) [7]	24	93	75	3	14
Pappas et al. (2008) [8]	22	100	91	29.5	—
Peregrin et al. (2008) [9]	55	88	65	36	25.5
Geddes et al. (2008) [10]	27	100	88.8	60	7.4
Valpreda et al. (2008) [11]	30	100	80	85.2	2.9
Polak et al. (2006) [12]	7	100	83.3	25.3	—
Audard et al. (2006) [13]	29	93.1	67.2	148	10.3
Salvadori et al. (2005) [14]	26	100	76.3	43.3	—
Voiculescu et al. (2005) [15]	31	90.3	75.5	45.2	12.9
Beecroft et al. (2004) [16]	17	100	94	27	10.5
Patel et al. (2001) [17]	17	94	82	26.9	—

*Estimated as significant clinical benefit or good graft function during the whole follow-up period.

**No procedure-related complication mentioned.
